# Leather Dyeing by Plant-Derived Colorants in the Presence of Natural Additives

**DOI:** 10.3390/ma15093326

**Published:** 2022-05-05

**Authors:** Patrycja Brudzyńska, Alina Sionkowska, Michel Grisel

**Affiliations:** 1 Department of Biomaterials and Cosmetics Chemistry, Faculty of Chemistry, Nicolaus Copernicus University in Torun, 87-100 Torun, Poland; alinas@umk.pl; 2 Chemistry Department, UNILEHAVRE, FR 3038 CNRS, URCOM EA3221, Normandie University, 76600 Le Havre, France; michel.grisel@univ-lehavre.fr

**Keywords:** leather dyeing, plant-derived colorants, natural coloration, chitosan

## Abstract

This research aimed to dye leather fabric samples with the application of plant-derived colorants and natural additives. Two grades of chitosan were used as additives, in addition to caffeine, nettle extract, and shellac solution. The ability of colorants to dye leather fabric and the impact of additives on leather fabric properties such as structure, color intensity, color stability under exposure to UVC irradiation, and mechanical properties were examined. For this purpose, dyed samples were tested by a colorimeter, ATR-FTIR spectrophotometer, mechanical testing machine, and X-ray diffractometer. The results indicated that the applied colorants of plant origin have the potential to dye leather fabrics without affecting their structure and without a negative impact on the environment. Applied natural additives can, therefore, beneficially influence the effects of the dyeing process, such as color intensity, colorfastness after exposure to UV irradiation, or tensile strength of the material.

## 1. Introduction

Leather is one of the commonly used materials in different industries to produce items such as clothes, shoes, bags, furniture, sports equipment, tools, and many other articles. Most popular are leathers obtained from animals such as cattle, goats, pigs, or sheep. Leather derived from mammals used in the production of numerous products is made from the central layer dermis, after removal of the two surrounding layers: the outer epidermis and the deepest layer, which is a subcutaneous fatty layer. Fresh leather contains water (60–70%) and protein (30–35%), which is mainly collagen [1,2,3]. In the leather preparation, it is necessary to remove nonfibrous protein and fat. This process, which is carried out using various chemicals such as salts, bases, acids, or enzymes, strengthens the bonds linking collagen fibers [4]. One of the most important parts of leather treatment converting this perishable material into a stable one is tanning. Tanning agents can be of vegetable or mineral origin, but animal oils can also be used [5]. Oil tanning is an old method, used since ancient times to soften the leather; dried pieces were placed in animal fats and then salted and smoked to preserve them. Many years later, ancients developed vegetable tanning with the use of plant parts including leaves, bark, and roots rich in tannin/tannic acid, thus explaining the origin of the process name [6]. Vegetable tanning still remains in use today; however, since the appearance of chemical tanning with the most commonly used chrome salts in the 19th century, its importance has diminished. Moreover, synthetic tanning agents containing hydrocarbons or phenols are also used. As there is a strong need to reduce the harmful influence of synthetic compounds used in production to tan or dye leather fabric, vegetable tannins and plant-derived colorants may gain importance. As examples, vegetable tanning agents issued from plants such as quebracho, mimosa, tara, and chestnut were examined, and their impact on leather properties was studied by Martins et al. [7]. Many researchers are focusing on replacing artificial and chemical compounds that are less beneficial for health and the environment with natural ones. Among others, Selvi et al. showed that leather fabric dyed with colorant extracted from *Bixa orellana* seeds indicated good coloring properties [8]. In another study, natural dye from *Cassia singueana* plant was successfully used to dye leather material with the addition of natural mordants [9]. Moreover, various attempts have been made to dye leather naturally. A few of these examples include dyeing leather with colorants obtained from *Coreopsis tinctoria* flower petals with the use of ultrasound and a magnetic stirrer [10], with lac dyes and the addition of mordants [11], with *Terminalia chebula retzius* improved by the enzymatic post-tanning process [12], with colorant obtained from sweet potato or black chokeberry, both with silver nanoparticles [13,14], and with indigo [15]. Research has shown that plant-derived colorants can efficiently dye leather fabrics. Moreover, leather fabric properties and the effects of the dyeing process can be improved by the application of different natural substances such as chitosan, which was examined by Burkinshaw and Karim [16] and Burkinshaw and Jarvis [17]. A substance of natural origin that can also improve fabric properties in terms of green finishing is shellac, which is also a source of lac dye traditionally used to color fabrics. This biopolymer resin derived from lac insect (*Kerria lacca*) secretion has the ability to stiffen, improve strength, and ensure water resistance, thus influencing the fabric’s longevity. Shellac is used in the leather industry to improve the shine, finish, and care of leather items. Likewise, shellac is used in sizing leather, e.g., in bookbinding practice. Research has shown that this compound affects not only leather fabrics; it was also proven that a chemically modified shellac finish can enhance the hydrophilicity and breaking strength of polyester material [18]. Other chemical compounds of plant origin, which may beneficially influence leather fabric dyeing and its color stability, are, for instance, caffeine and nettle extract. Due to their antioxidant activity and ability to protect against UV irradiation, they may have the potential to protect the leather from the photoaging process [19,20]. In the advanced fabric dyeing process, modern methods and techniques are also applied. Among others, ultrasonic treatment has been found as a sustainable and efficient solution for improving the color strength of plant-derived colorants, such as those isolated from Arjun bark [21] or Harmal seeds [22]. Research has shown that ultrasonic radiation not only influences the color of plant extract presenting various shades, but is also an environmentally friendly heating source, allowing the achievement of bright tints onto natural textiles. Microwave treatment effects were also studied on colorant extraction from Arjun bark [23] and coconut coir [24]. It was found that microwave rays had promising potential for dye extraction, resulting in darker shades on natural fabrics, and it may represent another ecofriendly tool for colorant isolation. Other advanced and sustainable dyeing systems based on radiation treatment include UV, gamma radiation, and plasma [25].

The aim of this study was to dye leather fabric samples with plant-derived colorant and apply various natural additives such as two grades of chitosan, caffeine, nettle extract, and shellac solution in the dyeing process. The purpose was to examine the ability of colorants to dye leather fabric and the impact of additives on leather fabric properties such as its structure, color intensity, color stability under exposure to UVC irradiation, and mechanical properties. Therefore, dyed samples were examined by a colorimeter, ATR-FTIR spectrophotometer, mechanical testing machine, and X-ray diffractometer, thus confirming the high potential of the tested dyeing solutions.

## 2. Materials and Methods

### 2.1. Materials

#### 2.1.1. Leather Fabric

**Figure 1:**
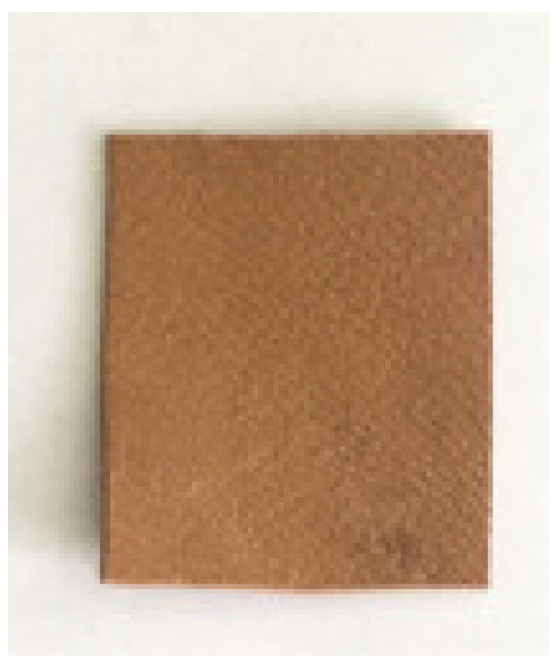
Undyed sample of vegetable tanned goat leather.

Undyed vegetable tanned goat leather purchased in Perskór (Radom, Poland) was used in the experiment (Figure 1).

#### 2.1.2. Colorants

**Figure 2:**
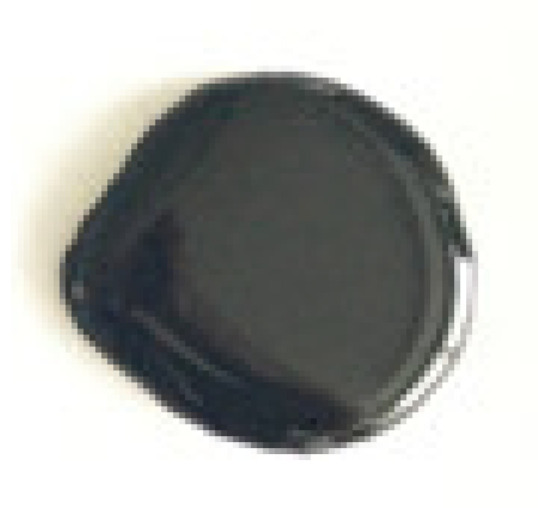
Purple food colorant of plant origin used in the experiment.

The leather fabric was dyed purple with liquid food colorant from EXBERRY^®^ by GNT (GNT Group B.V., Mierlo, The Netherlands), which was obtained via a physical manufacturing process with water (Figure 2).

#### 2.1.3. Additives Used in the Dyeing Process

For the dyeing process, two grades of chitosan were selected: low-molecular-weight chitosan powder from Sigma Aldrich (CAS number 9012-76-4, Darmstadt, Germany) and cosmetic-grade chitosan powder from Calaya (Złotów, Poland) natural cosmetic raw materials. In addition, liquid nettle extract containing water, *Urtica dioica* L. folium, and citric acid with a concentration of 30 g/L (EkaMedica) were used. Food-grade caffeine powder was purchased from Ting Trading Ltd. (Alfreton, United Kingdom) and food-grade rectified spirit (95%) was purchased from Polmos SA (Warsaw, Poland). Citric acid monohydrate pure P.A. was bought from Avantor Performance Materials Poland SA (CAS number 5949-29-1, Gliwice, Poland). Shellac flakes (blonde and dewaxed) were purchased in a local art restoration and conservation shop.

### 2.2. Methods

#### 2.2.1. Dyeing Process

Vegetable tanned goat leather was cold-dyed for 24 h in acidic conditions (liquor ratio 1:25). Purple colorant was dissolved in distilled water in concentrations of 5% (*w*/*w*). In addition to colorant, the following were added to the dyebath: caffeine (CAF) at a concentration of 2% (*w*/*w*) or caffeine (CAF) at a concentration of 2% (*w*/*w*) and nettle extract (concentration 30 g/L) instead of water. Samples were also pretreated with two grades of chitosan: 1% low-molecular-weight chitosan solution in 3% citric acid (*w*/*w*) (LMWCS/CA) or 1% cosmetic-grade chitosan solution in 3% citric acid (*w*/*w*) (CGCS/CA) for 1 h at room temperature in a 1:10 liquor ratio and then dyed. Moreover, the purple-dyed sample without additives was covered with a thin layer of shellac (SH) solution in ethanol at the concentration of 20% (*w*/*w*). To clarify the dyeing process of leather fabrics, all parameters are gathered in Table 1. Photos and microscope images (50× magnification, Motic Incorporation Ltd., Hong Kong, China) of undyed and purple-dyed leather samples are presented in Table 2 and Table 3.

#### 2.2.2. UV Irradiation of Fabrics

Undyed and purple-dyed leather fabrics were irradiated at a distance of 5 cm from the UV lamp emitting mainly UVC (ULTRAVIOL NBV 15, Ultra-Viol, Zgierz, Poland) for 30 min, 2 h, and 4 h with an intensity of radiation of 21.5 W/m^2^ at a wavelength of 254 nm.

#### 2.2.3. Colorimetric Measurements

Color measurements of dyed leather samples were performed using a Colorimeter CL 400, Courage, Khazaka, Köln, Germany (core measuring area: 5 mm; illuminated area: approximately 17 mm; range of emitted wavelengths: 440–670 nm). ΔE values were calculated on the basis of the mean values of three measurements of color parameters L*a*b (measurement uncertainty: ±5%) to study the color change after leather dyeing and after UVC irradiation (30 min, 2 h and 4 h), using Equation (1).

ΔE = (ΔL^2^ + Δa^2^ + Δb^2^)^0.5^, (1)

where ΔL = L − L_0_, Δa = a − a_0_, and Δb = b − b_0_; L_0_, a_0_, and b_0_ are values of reference samples.

#### 2.2.4. ATR-FTIR (Attenuated Total Reflection Fourier-Transform Infrared) Spectroscopy

For undyed and dyed leather fabric samples before and after UVC irradiation, ATR-FTIR spectra were obtained (the range of 400–4000 cm^−1^, absorption mode at 4 cm^−1^ intervals, 64 scans). Before any sample measurements, background scanning was performed. To record spectra, a Nicolet iS10 spectrophotometer with an ATR accessory and diamond crystal (Thermo Fisher Scientific, Waltham, MA, USA) was used, while, to process data, OMNIC software (Thermo Fisher Scientific, Waltham, MA, USA) was used.

#### 2.2.5. Mechanical Properties

A mechanical testing machine (Z.05, Zwick and Roell, Ulm, Germany) was used to examine the mechanical properties of leather fabric samples, which were cut in the shape of a rectangle (5 cm × 1 cm, five measurements). Testing program parameters were fixed as follows: the speed starting position was 50 mm/min, the speed of the initial force was 5 mm/min, and the initial force was 0.1 MPa. Data were collected using the TestXpert II 2017 program, and results were presented as average values with standard deviations (statistical analysis: Q-Dixon test).

#### 2.2.6. XRD

XRD patterns of leather fabric (before dyeing, after dyeing, and then after 4 h of UVC irradiation) were recorded using a Philips X”Pert with X’Celerator Scientific detector. The device parameters were as follows: CuKα radiation (λ = 1.541874 Å), anode voltage from 15 to 60 kV, anode current from 5 to 55 mA, slit collimator 1.0–1.0–0.1 nm, and angular rate of the scintillation meter 0.0010°/min. Match! software version 3.12 Build 214 was used to elaborate the obtained results.

## 3. Results

### 3.1. Colorimetric Measurements

ΔE values for undyed and purple-dyed leather samples are presented in Table 4 and Table 5. According to the ISO 11664-4:2019 standard, a color change is observed if the ΔE value is greater than 5. After exposure to UVC irradiation, the color of dyed leather fabric samples remain unchanged, except for the purple-dyed leather sample with caffeine addition after 4 h of UVC irradiation.

### 3.2. ATR-FTIR Spectroscopy

**Figure 3:**
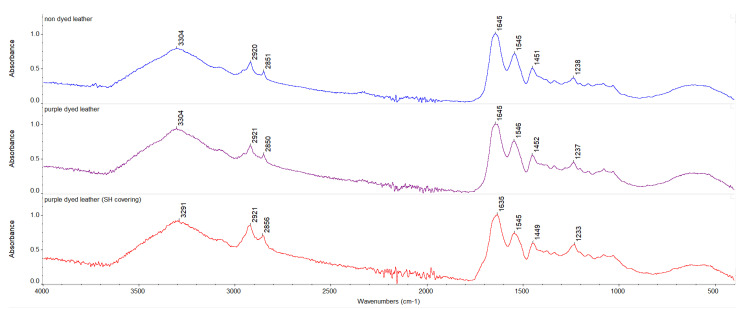
Infrared spectra (from the top) of undyed leather, purple-dyed leather, and purple-dyed leather with shellac covering from 4000 to 500 cm^−1^.

**Figure 4:**
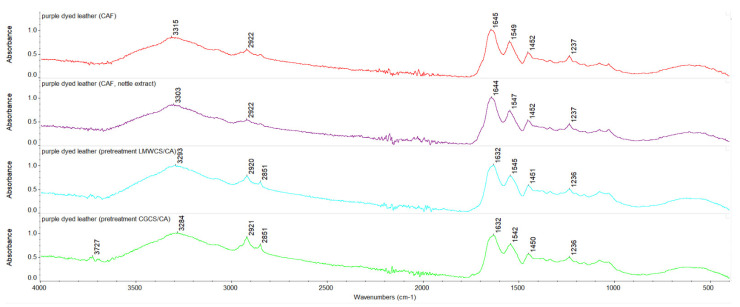
Infrared spectra (from the top) of purple-dyed leather with caffeine, purple-dyed leather with nettle extract and caffeine, purple-dyed leather pretreated with CGCS, and purple-dyed leather pretreated with LMWCS from 4000 to 500 cm^−1^.

Infrared spectra were registered for undyed and purple-dyed leather fabric samples with various additives before and after 30 min, 2 h, and 4 h of UVC irradiation. The ATR-FTIR spectra of undyed and dyed leather fabric samples before exposure to UVC irradiation are shown in Figure 3 and Figure 4.

**Figure 5:**
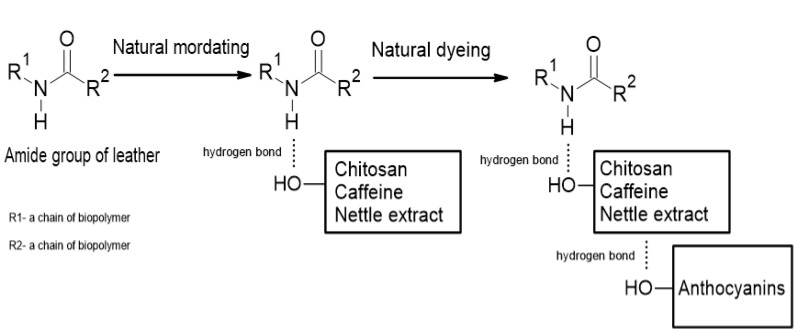
Scheme of proposed interactions of chitosan/caffeine/nettle extract-treated leather fabric dyed with plant-derived colorant containing anthocyanins.

In Table 6, wavenumbers of characteristic bands for leather fabrics are listed, and results for undyed and dyed leather fabric samples before and after UVC irradiation are gathered. Characteristic band positions for purple-dyed leather remained unchanged when compared to the undyed leather sample. For purple dyed samples with caffeine addition, the amide A band was shifted to higher wavenumbers, whereas, for purple-dyed samples pretreated with chitosan and for samples covered with shellac solution, the amide A band and amide I band were shifted to lower wavenumbers. Once exposed to UVC irradiation, changes in characteristic band position could be observed for the amide A band. The most noticeable changes were observed for purple-dyed leather samples with caffeine addition and with caffeine and nettle extract addition. For samples covered with shellac solution, changes were also observed. The position of the amide A band depends on the strength of the hydrogen bond formed by NH group, whereas the position of the amide I band depends on the strength of the hydrogen bond linking secondary structure elements. A scheme of the proposed interactions of chitosan/caffeine/nettle extract-treated leather fabric dyed with plant-derived colorant containing anthocyanins is presented in Figure 5.

### 3.3. Mechanical Properties

**Figure 6:**
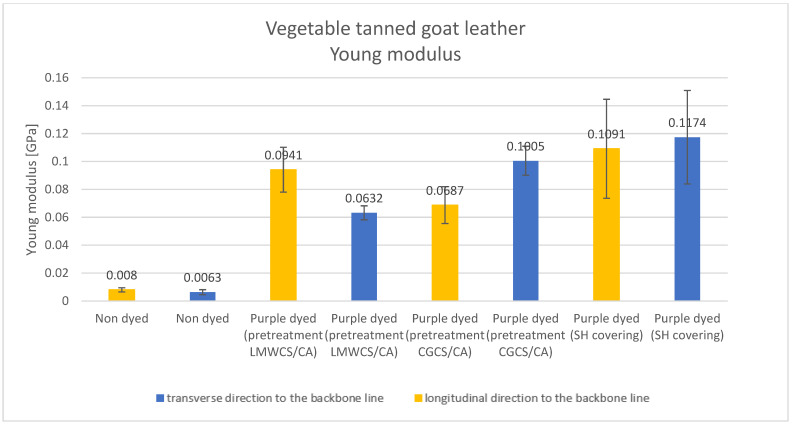
Young modulus parameter for undyed and dyed vegetable tanned goat leather fabrics.

**Figure 7:**
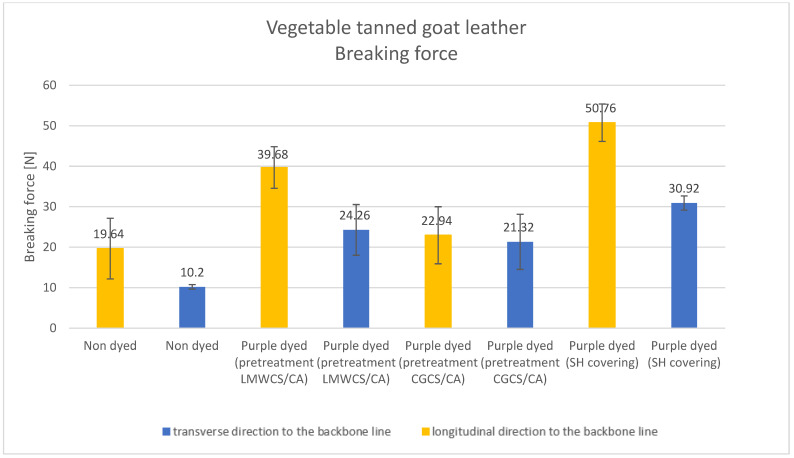
Breaking force parameter for undyed and dyed vegetable tanned goat leather.

**Figure 8:**
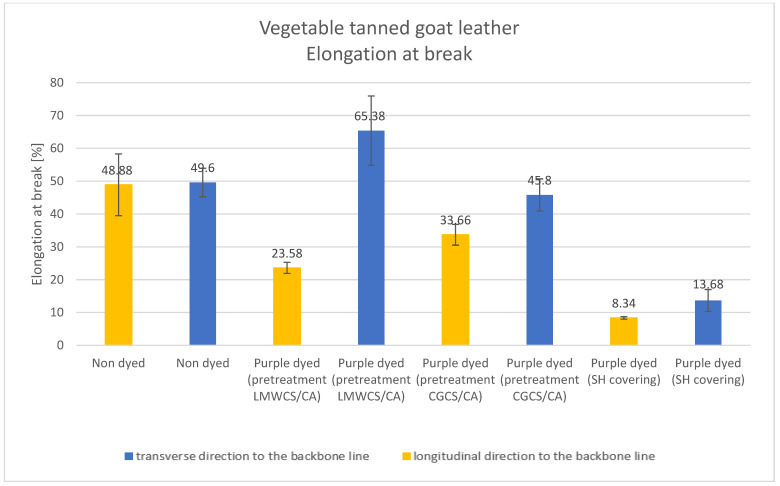
Elongation at break parameter for undyed and dyed vegetable tanned goat leather.

**Figure 9:**
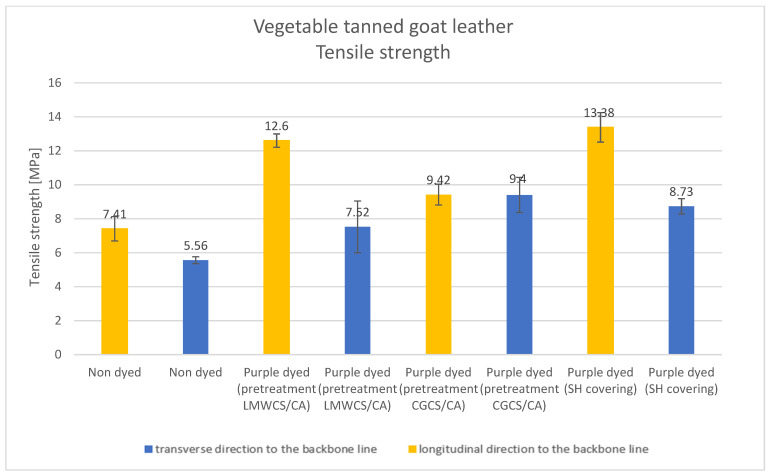
Tensile strength parameter for undyed and dyed vegetable tanned goat leather.

Results of the tested mechanical properties (Young modulus, breaking force, elongation at break, and tensile strength parameters) of undyed and purple-dyed leather samples are presented in Figure 6, Figure 7, Figure 8 and Figure 9.

Young modulus parameter values were the lowest for undyed vegetable tanned goat leather and the highest for the purple-dyed leather covered with shellac solution. There was no significant difference between values obtained along the transverse and longitudinal directions to the backbone for the undyed sample and purple-dyed sample covered with SH solution; in addition, differences could be observed for purple-dyed samples pretreated with chitosan. Values of the Young modulus parameter obtained for all dyed samples with different additives were significantly higher than those observed for undyed leather. The increase in Young modulus for the dyed samples may suggest that, after the dyeing process, new interactions appeared in the materials which influenced the mechanical properties. A similar trend was found for the breaking force of the materials after dyeing with purple colorant.

The breaking force parameter also showed the lowest values for undyed leather samples, while the highest was observed for the purple-dyed sample covered with SH solution. Values of this parameter obtained along the longitudinal direction to the backbone line were significantly higher than those obtained along the transverse direction; this was only also seen in the sample pretreated with CGCS. This fact clearly shows that the addition of SH and CGCS led to the modification of the mechanical properties. One of the important mechanical properties of leather fibers is elongation at break.

The lowest values of elongation at break were obtained for the purple-dyed leather sample covered with shellac solution, while the highest was obtained for the purple-dyed leather sample pretreated with LMWCS (transverse direction to the backbone line). It is known from our previous study that chitosan and collagen interact via hydrogen bonds, which may lead to an increase in the elongation at break parameter [26]. For the undyed sample, the values of elongation at break were very similar along both directions, while, for all dyed samples with various additives, differences were noticeable; in all measured samples, values of elongation at break were higher along the transverse direction to the backbone line. All values of this parameter were lower for leather samples dyed with different additives when compared to undyed samples, except for the purple-dyed sample pretreated with LMWCS along the transverse direction to the backbone line, for which the obtained value of this parameter was higher.

The tensile strength parameter had the lowest value for the undyed sample and the highest value for the purple-dyed leather with SH covering. Values of the tensile strength parameter were noticeably higher along the longitudinal direction to the backbone line; this was only also seen for the purple-dyed sample pretreated with CGCS.

### 3.4. XRD

**Figure 10:**
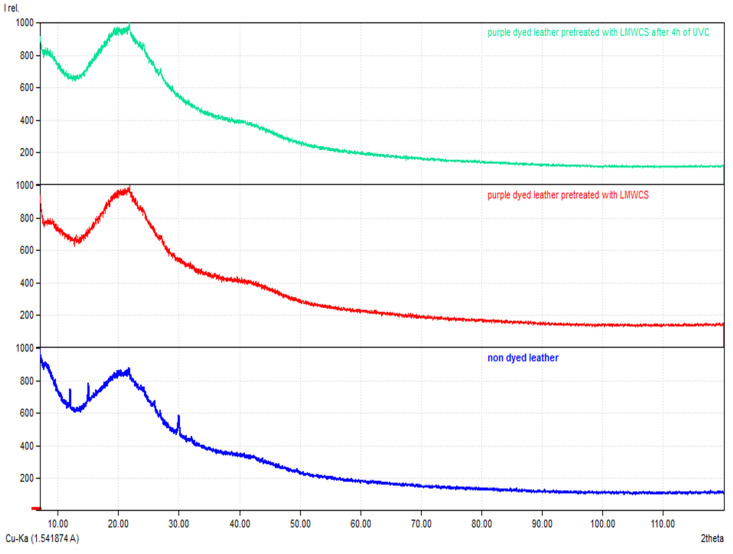
XRD spectra for undyed leather, purple-dyed leather pretreated with LMWCS, and purple-dyed leather pretreated with LMWCS after 4 h of UVC irradiation.

The XRD results of undyed leather and purple-dyed leather pretreated with low-molecular-weight chitosan before and after 4 h of UVC irradiation are presented below in Figure 10. The major diffraction peaks in all samples were at about 2θ = 8° (intermolecular lateral packing in collagen fibrils) and 20° (amorphous scattering, because of unordered collagen fibrils) [27]. In the undyed leather sample, there were three additional small peaks compared to the dyed and irradiated sample (2θ = 12°, 15°, and 30°), but these were probably artefacts. The XRD peak intensities for all samples were not greatly different; for dyed and for dyed then irradiated samples, the intensities of the peak at about 8° were reduced in comparison to the undyed sample, whereas the peak at about 20° appeared more intense. Thus, it seems that leather pretreatment before dyeing led to alterations of the ordered structure.

Previously, a link between tanning agent and increasing helical rise per residue distribution was found, but a direct link with the tanning agent concentration was not clear [27]. Leather pretreatment before dyeing led to some alterations, but its final influence on XRD spectra was probably negligible.

## 4. Discussion

The purple colorant of plant origin efficiently dyed the vegetable tanned goat leather. The applied additives in the dyeing process caused a greater color change as illustrated by the increase in ΔE parameter, thus dying the leather more intensely than when only colorant was used. Caffeine and nettle extract addition, low-molecular-weight chitosan pretreatment, and shellac covering significantly improve colorfastness under the influence of UVC irradiation; nevertheless, values of ΔE parameters were lower than for the purple-dyed sample; thus, a lesser color change was observed. Dyeing and pretreatment with chitosan and shellac covering resulted in a greater tensile strength of the examined leather samples. ATR-FTIR spectra indicated that the dyeing process did not affect the structure of the leather fabric; however, the shift of the amide bands indicated the presence of interactions between leather and chitosan.

Electrostatic interactions or hydrogen bonding between collagen and chitosan influence complex creation. Because of chitosan’s cationic properties, interactions with the anionic part of collagen are possible [28]; moreover, chitosan also makes the collagen structure more stable [29]. However, the interaction between collagen and chitosan molecules is weak due to the long chain of collagen molecules and low water solubility; hence, there is a need to add functional groups with higher activity to improve molecule binding [30]. Chitosan’s impact on the collagen structure and, thus, on leather properties is not fully explained. It is essential to study the interaction between chitosan and collagen, because it can determine the properties of finished leather [30]. Collagen molecules also interact with polyphenols present in plant extracts; the stability of these complexes depends on the strength of interaction, but is also connected to the distribution of polyphenolic molecules in the collagen structure [31]. Natural dyed fabrics may have insufficient fastness to light. Leather dyed with natural colorants with and without mordants was investigated for light fastness. Interestingly, the study showed that dyed leather indicated high fastness to light, which can be explained by the interaction between natural dyes and leather proteins, which protect natural colorants from the oxidation process [32].

The environmental regulations and compliance regarding leather tanning and leather dyeing have compelled the leather industry to seek alternative cleaner ingredients with the capacity to minimize or prevent pollution caused by hazardous chemicals [33]. It is expected that the production of leather materials is possible by replacing the current use of synthetic chemicals such as chromium salts, dyes, fat liquors, and surfactants or minimizing their usage by incorporating agro-based organic components. The review paper by Nalyanya et al. [33] showed that there are striking similarities in the leather properties of leather processed using natural plants and using synthetic chemicals. The trends and opportunities for future research in sustainability research in the leather industry were recently summarized by Omoloso et al. [4]. Sustainability research continues to receive significant attention in academia, in the service and manufacturing industries, and in the leather manufacturing industry. There are also new routes to dye leather fabrics. Certain species of filamentous fungi typically produce colored substances as secondary metabolites, which can be used as dyes for industrial applications. As a consequence, such natural biodyes can be envisaged as ecofriendly alternatives to synthetic dyes since they do not originate from extractive activities of the environment and no hazardous chemicals are used during their production. A review of the recent literature regarding improvements of leather dyeing techniques and the search of natural dyes for industrial uses, emphasizing the developments related to dyes from filamentous fungi *Monascus purpureus*, was published by Fuck et al. [34]. Although there are many possibilities to dye leather materials for everyday use, the most important is the final destination of dyed leather. Lastly, the selection of dye for dyeing leather strongly depends on the nature of the tonnage and the demands of the dyeing (surface dyeing, dyeing with deep penetration, etc.) [35].

## 5. Conclusions

Colorants of plant origin have the potential to dye leather fabrics with a negligible effect on their structure and no negative impact on the environment. The application of various natural additives such as chitosan, caffeine, nettle extract and shellac solution in the dyeing process can beneficially influence the dyeing process and properties of dyed fabrics, such as color intensity, colorfastness after exposure to UV irradiation, or tensile strength of the material. The interactions between the additives and leather are mainly via hydrogen bonds. Further research is needed to develop the dyeing process using natural colorants and improve the parameters of dyed leather fabrics.

## Figures and Tables

**Table 1:** Parameters of dyeing process of leather fabric samples.

Type of fabric	Vegetable tanned goat leather					
Liquor ratio	1:25					
Type of dyeing process	Cold-dyeing					
Color of dye	Purple					
Dye concentration (%)	5					
pH of dyebath	3.30	3.30	3.33	3.27	3.30	3.20
Pretreatment before dyeing	no	no	no	no	LMWCS/CA	CGCS/CA
Additives in dyeing process	no	SH covering	CAF	CAF/nettle extract	no	no

**Table 2:** Photos of undyed and purple-dyed vegetable tanned goat leather fabrics samples.

Undyed vegetable tanned goat leather					
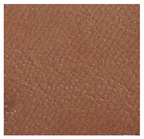					
Dyed vegetable tanned goat leather					
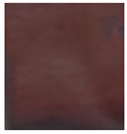	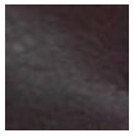	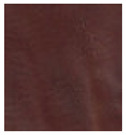	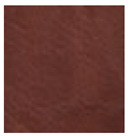	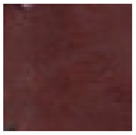	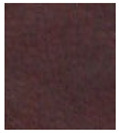
Purple colorant (5%)	Purple colorant (5%)/SH covering	Purple colorant (5%)/CAF addition	Purple colorant (5%)/CAF and nettle extract addition	Purple colorant (5%)/LMWCS pretreatment	Purple colorant (5%)/CGCS pretreatment

**Table 3:** Microscope images (50× magnification) of undyed and purple-dyed leather fabric samples.

Undyed vegetable tanned goat leather					
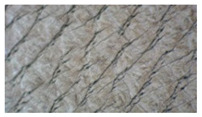					
Dyed vegetable tanned goat leather					
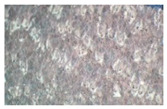	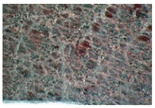	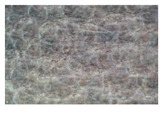	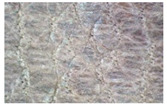	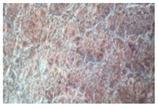	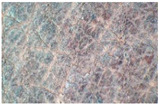
Purple colorant (5%)	Purple colorant (5%)/SH covering	Purple colorant (5%)/CAF addition	Purple colorant (5%)/CAF and nettle extract addition	Purple colorant (5%)/LMWCS pretreatment	Purple colorant (5%)/CGCS pretreatment

**Table 4:** Values of L*a*b and ΔE parameters for leather fabric samples after dyeing with colorant of plant origin and additives (mean values of three measurements; measurement uncertainty ±5%).

Type of fabric	Vegetable tanned goat leather						
Color of dye	Non dyed	Purple					
Dye concentration (%)	-	5					
Pretreatment before dyeing	-	no		no	no	LMWCS/CA	CGCS/CA
Additives in dyeing process	-	no		CAF	CAF/nettle extract	no	no
pH of dyebath	-	3.30		3.33	3.27	3.30	3.20
SH covering	-	No	Yes	No	No	No	No
Values of parameter L	48.76	36.45	29.31	31.29	34.57	34.93	28.59
Values of parameter a	10.98	8.91	0.78	6.33	9.63	7.72	5.62
Values of parameter b	23.72	13.24	7.16	11.95	15.51	10.98	12.04
ΔE after dyeing	-	16.30	27.51	21.57	16.45	19.08	23.92

**Table 5:** ΔE parameter values for undyed and purple-dyed leather fabric samples before and after UVC radiation (mean values of three measurements; measurement uncertainty ±5%).

Type of fabric	Vegetable tanned goat leather						
Color of dye	Non dyed	Purple					
Dye concentration (%)	-	5					
Pretreatment before dyeing	-	no		no	no	LMWCS/CA	CGCS/CA
Additives in dyeing process	-	no		CAF	CAF/nettle extract	no	no
pH of dyebath	-	3.30		3.33	3.27	3.30	3.20
SH covering	-	No	Yes	No	No	No	No
ΔE (after 0.5 h of irradiation)	0.50	1.17	0.67	0.96	1.64	1.22	4.26
ΔE (after 2 h of irradiation)	0.75	2.21	0.30	1.91	2.12	1.91	2.64
ΔE (after 4 h of irradiation)	1.35	3.36	0.78	5.27	3.30	2.51	2.87

**Table 6:** Wavenumbers for characteristic band positions of undyed and purple-dyed leather fabrics before and after UVC irradiation.

Sample	Time of UVC Radiation	Characteristic Bands (cm^−1^)								
		Amide A (N–H Stretching)	C–H Stretching	Amide I (C=O Stretching)	Amide II (C–N Stretching, N–H Bending)	CH_2_ Bending of Proline	CH_2_ Stretching, C–H Bending	Amide III (C–N Stretching, N–H Bending)	C–OH Stretching	C–O, C–O–C Stretching
Undyed leather	0 h	3304	2920/2851	1645	1545	1451	1338	1238	1161	1082
	0.5 h	3298	2918/2850	1650	1545	1453	1337	1238	1163	1083
	2 h	3304	2920/2851	1646	1542	1452	1338	1237	1162	1082
	4 h	3308	2922/2851	1647	1544	1452	1338	1237	1162	1084
Purple-dyed leather	0 h	3304	2921/2850	1645	1546	1452	1338	1237	1162	1080/1032
	0.5 h	3293	2922/2851	1640	1547	1451	1338	1236	1162	1081/1032
	2 h	3304	2921/2852	1645	1548	1451	1337	1237	1162	1081/1033
	4 h	3299	2922/2852	1645	1545	1450	1337	1236	1162	1080/1032
Purple-dyed leather (SH covering)	0 h	3291	2921/2856	1634	1545	1449	1336	1233	1161	1080/1031
	0.5 h	3305	2924/2854	1632	1542	1447	1337	1234	1162	1075/1039
	2 h	3310	2923/2853	1634	1542	1450	1339	1234	1162	1079/1040
	4 h	3306	2924/2851	1639	1542	1448	1338	1234	1163	1077/1033
Purple-dyed leather (CAF)	0 h	3315	2922/2849	1645	1549	1452	1338	1237	1163	1079/1032
	0.5 h	3294	2921/2850	1644	1548	1450	1337	1237	1163	1080/1031
	2 h	3308	2921/2852	1645	1549	1449	1337	1237	1163	1080/1031
	4 h	3307	2922/2853	1645	1548	1450	1337	1238	1163	1081/1030
Purple-dyed leather (CAF, nettle extract)	0 h	3303	2922/2850	1644	1547	1452	1339	1237	1163	1079/1031
	0.5 h	3299	2918/2849	1639	1546	1451	1335	1236	1163	1080/1031
	2 h	3288	2920/2853	1644	1549	1450	1336	1236	1162	1079/1030
	4 h	3297	2924/2853	1645	1550	1450	1337	1237	1161	1080/1031
Purple-dyed leather (pre-treatment LMWCS/CA)	0 h	3293	2920/2851	1632	1545	1451	1337	1236	1160	1079
	0.5 h	3281	2918/2850	1632	1545	1449	1336	1234	1157	1078
	2 h	3287	2919/2851	1634	1542	1448	1337	1235	1161	1079
	4 h	3291	2923/2852	1634	1545	1447	1338	1236	1161	1078
Purple-dyed leather (pre-treatment CGCS/CA)	0 h	3284	2921/2851	1632	1542	1450	1338	1236	1160	1080
	0.5 h	3277	2917/2850	1631	1545	1450	1337	1234	1161	1079
	2 h	3288	2929/2851	1633	1542	1448	1337	1236	1159	1079
	4 h	3289	2923/2853	1630	1543	1448	1338	1235	1159	1078

## Data Availability

Not applicable.
